# Supplementary material for: The interactome of intact mitochondria by cross-linking mass spectrometry provides evidence for coexisting respiratory supercomplexes
Source: Mol Cell Proteomics. 2017 Dec 8;17(2):216–32. doi: 10.1074/mcp.RA117.000470 (PMC5795388; doi:10.1074/mcp.RA117.000470)
Supplement: Supplemental Data [file supp_RA117.000470_133922_0_supp_23978_fzffwf.zip › spectra_annotation/mito_spectra_annotation/272-1-7-1-9-1.pdf]

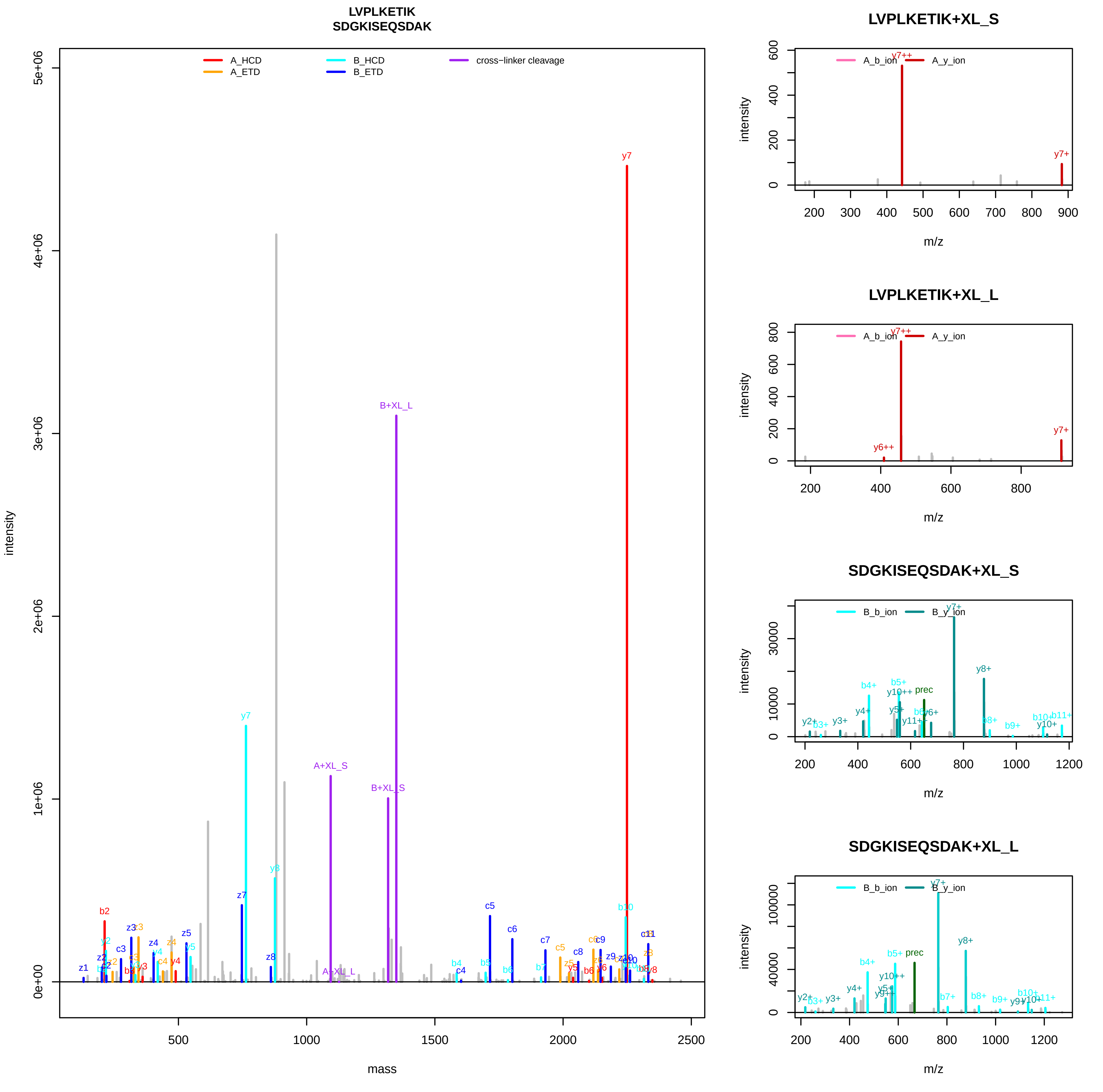
LVPLKETIK
SDGKISEQSDAK
A_HCD
A_ETD
B_HCD
B_ETD
cross−linker cleavage
intensity
mass
y7
B+XL_L
A+XL_S
B+XL_S
A+XL_L
y8
z7
b2
b10
c5
c6
c7
c8
c9
c10
c11
z3
z4
z5
z8
z9
z10
y5
y4
c3
c4
b4
b5
b6
b7
b8
0e+00
1e+06
2e+06
3e+06
4e+06
5e+06
500
1000
1500
2000
2500
LVPLKETIK+XL_S
A_b_ion
A_y_ion
y7++
y7+
m/z
LVPLKETIK+XL_L
y6++
SDGKISEQSDAK+XL_S
B_b_ion
B_y_ion
y7+
y8+
b4+
b5+
y10++
prec
y6+
y11++
y2+
b3+
y3+
y4+
y5+
b8+
b9+
b10+
b11+
y10+
SDGKISEQSDAK+XL_L
b7+
y9++
y9+
